# A Glance at Processing-Microstructure-Property Relationships for Magnetoelectric Particulate PZT-CFO Composites

**DOI:** 10.3390/ma13112592

**Published:** 2020-06-06

**Authors:** Pietro Galizia, Carlo Baldisserri, Elisa Mercadelli, Claudio Capiani, Carmen Galassi, Miguel Algueró

**Affiliations:** 1 Institute of Science and Technology for Ceramics (ISTEC), CNR, I-48018 Faenza, Italy; pietro.galizia@istec.cnr.it (P.G.); carlo.baldisserri@istec.cnr.it (C.B.); claudio.capiani@istec.cnr.it (C.C.); carmen.galassi@istec.cnr.it (C.G.); 2 Instituto de Ciencia de Materiales de Madrid (ICMM), CSIC, Cantoblanco, 28049 Madrid, Spain; malguero@icmm.csic.es

**Keywords:** cobalt ferrite, particulate composite, magnetoelectric coupling, piezoelectric, dielectric, grain-size effect, Sauter’s diameter, tetragonality, lattice parameters, poling

## Abstract

In this work, we investigated the processing-microstructure-property relationships for magnetoelectric (ME) particulate composites consisting of hard ferromagnetic CoFe_2_O_4_ (CFO) particles dispersed in a Nb-doped PbZr_x_Ti_1-x_O_3_ (PZT) soft ferroelectric matrix. Several preparation steps, namely PZT powder calcination, PZT-CFO mixture milling and composite sintering were tailored and a range of microstructures was obtained. These included open and closed porosities up to full densification, PZT matrices with decreasing grain size across the submicron range down to the nanoscale and well dispersed CFO particles with bimodal size distributions consisting of submicron and micron sized components with varying weights. All samples could be poled under a fixed DC electric field of 4 kV/mm and the dielectric, piezoelectric and elastic coefficients were obtained and are discussed in relation to the microstructure. Remarkably, materials with nanostructured PZT matrices and open porosity showed piezoelectric charge coefficients comparable with fully dense composites with coarsened microstructure and larger voltage coefficients. Besides, the piezoelectric response of dense materials increased with the size of the CFO particles. This suggests a role of the conductive magnetic inclusions in promoting poling. Magnetoelectric coefficients were obtained and are discussed in relation to densification, piezoelectric matrix microstructure and particle size of the magnetic component. The largest magnetoelectric coefficient α_33_ of 1.37 mV cm^−1^ Oe^−1^ was obtained for submicron sized CFO particles, when closed porosity was reached, even if PZT grain size remained in the nanoscale.

## 1. Introduction

The increase of the magnetoelectric (ME) response of multiferroic composite systems up to tens of V/cm·Oe has turned them into attractive candidates for the development of novel devices [1,2,3,4,5]. Potential applications include broadband magnetic field sensors [6,7,8,9,10,11], multi-state memories [12,13,14], energy harvesting devices [15,16,17,18,19,20] phase shifters [21,22,23], diodes [19], inductors [4] and ME coupled memristors [24,25]. Up to the present time, the highest magnetoelectric coefficients have been achieved by combining a ferroelectric phase with high piezoelectric response and a ferromagnetic phase with large magnetostriction through strain mediation. Among ferroelectric phases, pure or doped PbZr_x_Ti_1-x_O_3_ (PZT) perovskite compounds close to the morphotropic phase boundary (MPB) are frequently employed due to their very high dielectric constant and electromechanical coupling [26,27,28,29,30,31,32,33]. As to the magnetostrictive phase, CoFe_2_O_4_ (cobalt ferrite, CF) spinel oxide is a very interesting component although its large magneto-crystalline anisotropy limits its magnetic field sensitivity, that is, the slope of the magnetostriction curve vs. applied magnetic field [34,35]. Full poling of the piezoelectric phase is required, which is easily attained in laminated ME composites [36,37]. Such composites can be effectively poled because of the separation between the insulating piezoelectric and low-resistivity magnetostrictive phases associated with the 2-2 connectivity. However, for particulate composites where spinel grains are dispersed within the perovskite matrix (0-3 connectivity), the electric resistivity of the material is reduced due to the non-negligible conductivity of the magnetic phase. As a result, non-saturated hysteresis loops are evident in all particulate ME composites [19,30,38,39]. Actually, uncontrolled partial poling is likely behind the large variability in ME responses. Namely, studies on the effect of the of CFO/PZT volume fraction (x) found optimal compositions that span across the interval 0.15 < x < 0.4 of CFO [28,40,41,42], while theoretical studies predict a maximum of response for x = 0.5. Composite density decreases continuously but not linearly, with increasing amount of the CFO fraction with lower density, showing a decrease of 10% between 0 and 0.39 of CFO [42]. The problem is that densification becomes increasingly difficult even at moderate CFO contents. This affects composite permittivity (ε_r_) and charge piezoelectric coefficients (d_33_) which can show a drastic decrease up to 60% and 80% respectively, even at only 0.14 CFO volume fraction. Although by avoiding PbO loss and side reactions full densification was achieved even for 0.274 CFO [28,43], a d_33_ piezoelectric coefficient of only 30 pC/N was measured instead of 340 pC/N, the latter being typical of fine-grained soft PZT [19,44].

In the present study, we report the dielectric, piezoelectric, elastic and magnetoelectric properties of CFO/PZT particulate composites having a fixed volume content of CFO (x = 0.144) but a wide range of microstructures. Densification values from 100 down to 62% were achieved, as well as grain size distributions of both phases covering the micron, submicron and nanometer ranges. This was done by varying the calcination temperature for PZT, the mixing/milling process of CFO and PZT powders and the sintering cycle. The focus was placed on the relationships among preparation, microstructure and properties (i.e. dielectric, mechanical and piezoelectric constants and electromechanical conversion factors) and on how magnetoelectric coefficients depend on microstructure. Objective is to shed light into the origin of the wide scatter of values found in the literature for particulate composites of a given material system with fixed phase fractions and of the discrepancies between experimental coefficients and those expected in the theoretical studies. Additionally, most favorable microstructures are identified and procedures to obtain them are presented. This work has manifold implications because it shows how to tune the magnetoelectric response by the processing/microstructure and gives some guidelines to increase the magnetoelectric coefficient while keeping constant the CFO content. 

## 2. Materials and Methods

### 2.1. Sample Preparation

Composites of the fixed composition 0.135CoFe_2_O_4_-0.865Pb_0.988_(Zr_0.52_Ti_0.48_)_0.976_Nb_0.024_O_3_ (CFO/PZT 14.4/85.6 vol%) were produced by two-step solid-state-reaction. Firstly, perovskite PZT was synthesized starting from PbO (Sigma-Aldrich, Inc. Darmstadt, Germany), ZrO_2_ (SC 101, Mel Chemicals, Flemington, NJ, USA), TiO_2_ (P 25, Evonik Degussa, Essen, Germany), Nb_2_O_5_ (Sigma-Aldrich, Inc. Darmstadt, Germany). A sequence of ball milling for 48 h, calcination at 850 °C for 4 h and further ball milling for 96 h in ethanol (powder P, as it is only partially converted to the perovskite phase) was followed [45]. The same powder was then re-calcined at 930 °C for 2 h (powder F, fully reacted to the perovskite phase). The spinel CFO powder was prepared as described in a previous work [46], by solid state reaction of nanosized cobalt oxide powder (Co_3_O_4_, Sigma-Aldrich, Inc. Darmstadt, Germany) and nanosized iron oxide (Fe_2_O_3_, Sigma-Aldrich, Inc. Darmstadt, Germany) at 850 °C for 2 h, followed by planetary milling. 

In order to increase the CFO dispersion and the reactivity of both phases, the CFO/PZT14.4/85.6 powder mixture was planetary milled (Pulverisette 6, Fritsch, Idar-Oberstein, Germany) in ethanol in a stainless steel jar filled to 75 vol.% with zirconia grinding balls (Ø = 1 mm, grinding balls/powder mass ratio equal to 9:1). One batch was milled at 350 rpm for 2 h (6 steps, 20 min each), which will be referred to as mild milling (M) and one batch was milled at 400 rpm for 10 h (30 steps, 20 min each) (strong milling, S). The milled powder mixtures were then cold-consolidated into discs of 12 mm diameter by die pressing at 100 MPa, followed by cold isostatic pressing at 300 MPa. Finally, the green homogeneous CFO/PZT bodies were sintered in a Nannetti Kiln FCN 16 furnace controlled by an Ero-Electronic PKP controller. The sintering treatments were performed in lead-saturated atmosphere under different conditions, that is, at 1100 °C and 1150 °C for 2 h and heating rate of 2.5 °C/min (slow sintering, s) and at 1100 °C and 1150 °C for 1 min and heating rate of 44 °C/min (fast sintering, f). Samples were brought back to room temperature by natural cooling of the furnace (in the case of fast sintering the temperature dropped to 800 °C in 4 min). The sintered pellets were then ground and electroded on both sides using Ag paste, thermally treated at 750 °C for 15 min and finally poled in silicone oil at 120 °C for 30 min under an applied DC field of 4 kV/mm. This was the maximum field all samples withstood before dielectric breakdown. Although several ones could resist higher fields, saturation was not attained in these cases either. Therefore, we chose to compare results at the same field.

### 2.2. Microstructural Characterization

The relative density of the sintered samples was calculated as the ratio between the experimental density determined by the Archimedes’ method and the composite theoretical density (7.618 g cm^−3^) calculated as the weighted average of PZT (8.006 g cm^−3^) and CFO (5.304 g cm^−3^) crystallographic densities.

The crystalline phases were identified by X-ray powder diffraction using a Bruker D8 Advance X-ray diffractometer (θ-θ equipped with a LINXEYE detector (Bruker, Karlsruhe, Germany), using Cu K_α_ radiation. Patterns were recorded in the 15° ≤ 2θ ≤ 80° range with 2.4°/min scanning rate.

The microstructure of the sintered samples was investigated by scanning electron microscopy (SEM-FEG, Carl Zeiss Sigma NTS GmbH, Oberkochen, Germany), embedding the cross sections in epoxy resin and then polishing them down to 0.25 µm finish. The grain size distributions of the sintered samples were calculated via image analysis of the SEM micrographs using ImageJ software (Java, ORACLE, Redwood City, CA, USA). 

### 2.3. Electrical Characterization and Magnetoelectric Response

Silver-electroded flat-disk samples (thickness-to-diameter ratio < 0.1) were dielectrically, piezoelectrically and mechanically characterized after poling, by performing frequency sweeps on an HP 4194A frequency response analyzer, (Hewlett-Packard Palo Alto, CA, USA), noting relevant resonance and anti-resonance peak frequencies both in the radial and thickness modes. The first resonance and anti-resonance and the second resonance frequency of the radial mode were measured, along with the first resonance and anti-resonance frequency of the thickness mode. All frequency values were measured with a precision better than 10^−4^. Dielectric and piezoelectric constants of the materials were then calculated based on the recorded frequency values and the measured values of the sample geometrical parameters and density. All calculations were performed in accordance with the ANSI/IEEE Standard 176–1987 using a MATLAB application (MathWorks, Natick, MA, USA). Values of the d_33_ piezoelectric coefficient were measured independently using a d_33_-meter (SinoCera, Sinoceramics, Shanghai China) (± 1 pC/N resolution) whose readings were preliminarily calibrated using a 360 pC/N standard sample provided by the manufacturer.

The magnetoelectric response of the composites was also characterized. A system comprising a combination of two Helmholtz coils, designed to independently provide a static magnetic field up to 1 kOe to magnetize the material and an alternate magnetic field of 10 Oe at 1 kHz (which acts as stimulus) was used, while the magnetoelectric voltage response was monitored with a lock-in amplifier. The 3-3 geometry was selected to obtain the α_33_^E^ longitudinal magnetoelectric coefficient as a function of the bias magnetic field H, after normalization to the ceramic element thickness.

## 3. Results and Discussion

### 3.1. Microstructure

**Figure 1:**
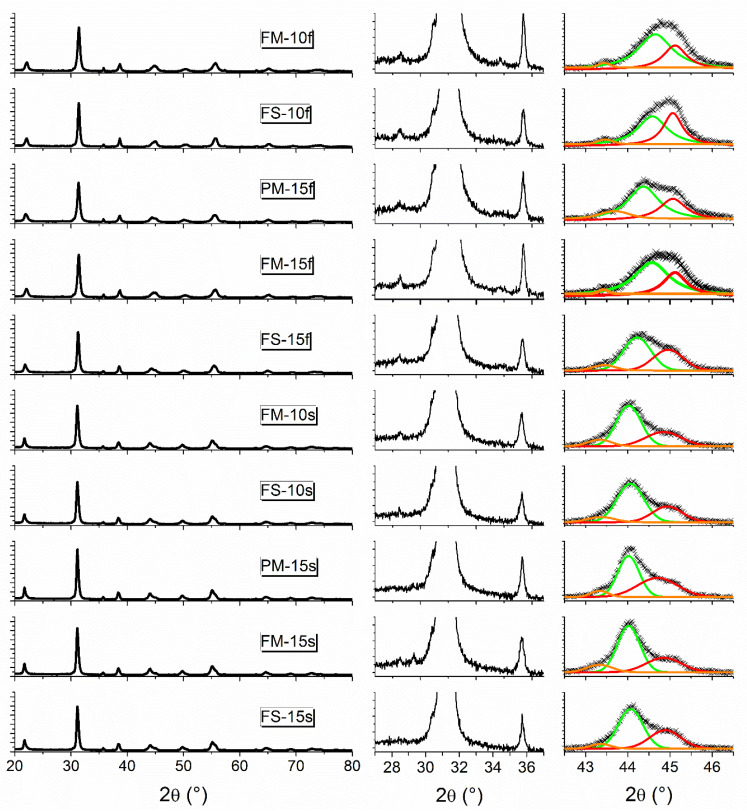
X-ray diffraction (XRD) patterns of the sintered composites. In all abscissae and ordinates 2θ values in degrees and cps are shown. In the first column, the entire diffractogram is shown (cps = 0–13000). The second column details the region with the highest peaks attributed to ZrO_2_ (2θ ≈ 28°), PZT (2θ ≈ 31°) and CFO (2θ ≈ 35.5°) (cps = 0–1100). In the third column, (0 0 2) and (2 0 0) diffraction peaks of the PZT tetragonal phase are fitted with solid orange and red lines, (2 0 0) peak of the PZT rhombohedral phase is fitted with a green line (cps = 0–1800). (For interpretation of the references to color in this figure legend, the reader is referred to the web version of this article).

Table 1 summarizes all the results from microstructural analysis. Samples sintered using slow heating rate achieved higher density values than those obtained by the fast sintering process. A range of values from 62% up to full densification were attained. As confirmed by XRD patterns (Figure 1), composites consisted in a mixture of perovskite PZT and spinel CFO (first column of Figure 1). Traces of ZrO_2_ were clearly detectable in all samples, except for PM-15s and FS-15s, although amounts below the technique sensitivity cannot be excluded. Related perovskite tiny compositional deviations would have a significant effect in properties only if they result in materials leaving the morphotropic phase boundary (MPB), which does not seem to be the case.

Actually, the splitting of the perovskite cubic 200 peak into three peaks between 42.5 and 46.6° indicates the coexistence of tetragonal (T) and rhombohedral (R) phases and thus, that the PZT phase was consistently within the MPB region [47,48,49,50]. As said, this is required for high piezoelectric response. Heating rate also influenced microstructure and submicron grain sizes were obtained by slow heating, while nano-structuring resulted from fast sintering. Slow sintering was then observed to result in a larger grain size with an apparent effect on crystal structure; the a-axis of the rhombohedral phase was found to systematically increase as grain size increased. Regarding the tetragonal phase, the c/a ratio was slightly larger in fast sintered materials, in spite of grain size being at the nanoscale. This might be an effect of the open porosity (and of the relaxation of the ceramic stress field) but also a compositional effect associated with a slightly increased ZrO_2_ amount, as suggested by the concomitance of higher ZrO_2_ peak and lower density [28]. Nevertheless, fast sintered composites also show coexistence of T and R perovskite phases and are still at the MPB. Therefore, a significant reduction of the piezoelectric response is not expected.

**Figure 2:**
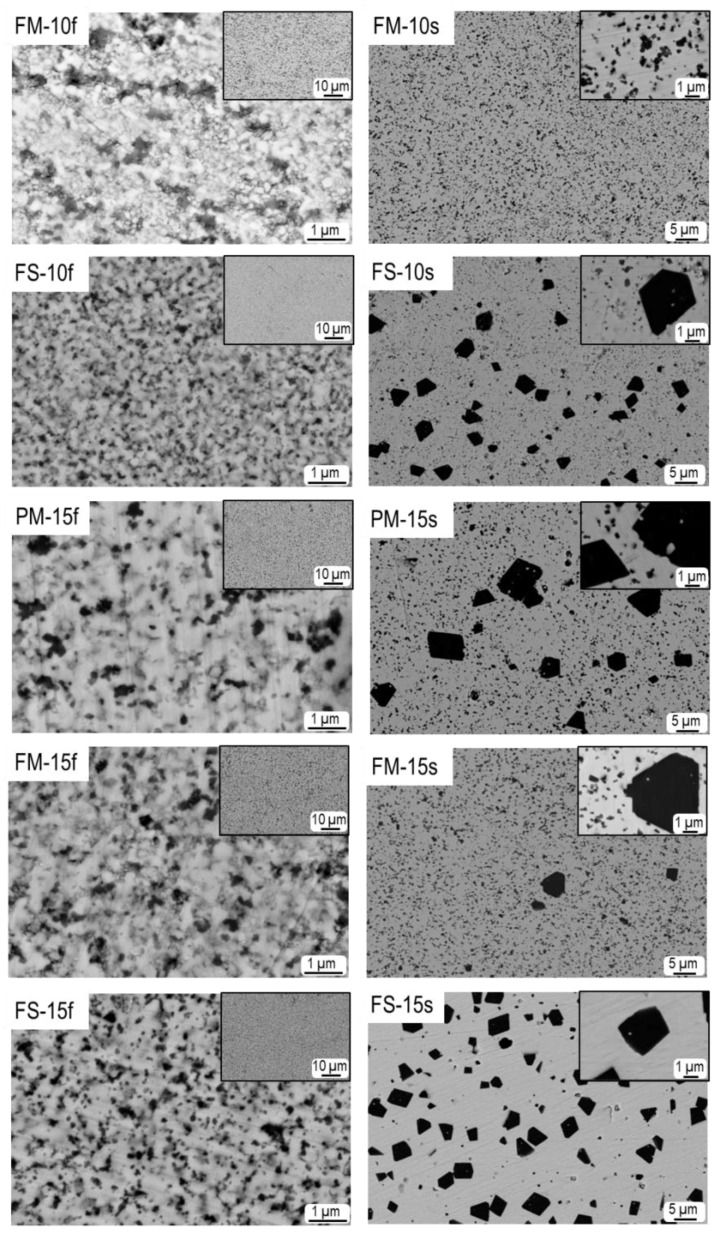
Back-scattered scanning electron microscopy (SEM) images of polished cross sections of the sintered samples.

Electron micrographs of polished cross sections showing the magnetostrictive component and its spatial distribution are given in Figure 2. Densification trends were confirmed and good dispersion of the magnetic particles was attained. Note the presence of unimodal and bimodal distributions of CFO particles with sizes within the submicron and micron ranges, respectively, depending on the sintering conditions. Bimodal distributions were observed in conventionally sintered (s) samples, with varying relative weights, from almost unimodal submicrometer size distribution for FM-10s, to a maximum fraction of micrometer-sized CFO grains for FS-15s. In agreement with our previous work on the grain growth of CFO [46], large CFO grains were obtained when strong milling conditions were employed during mixing. 

**Figure 3:**
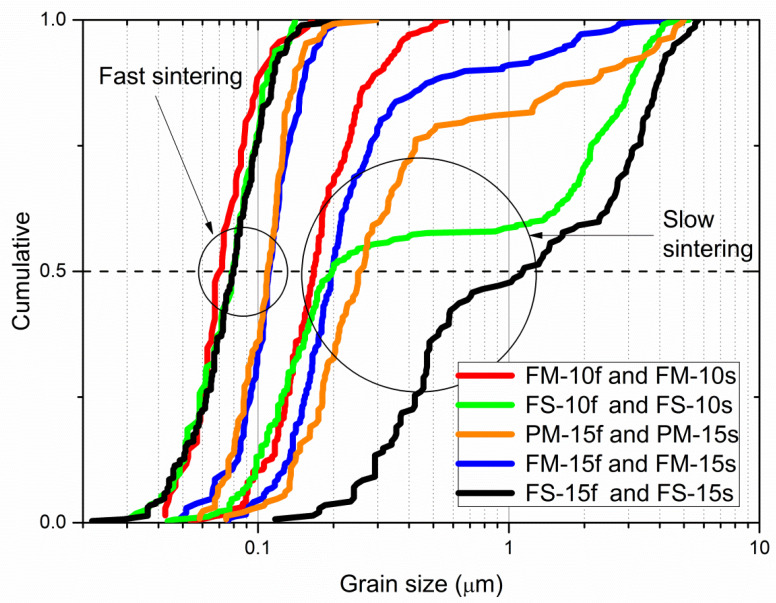
Cumulative grain size distribution curves of CFO measured on the electron micrographs of polished cross section of sintered samples. (For interpretation of the references to color in this figure legend, the reader is referred to the web version of this article.).

Cumulative frequencies of the CFO grain size are shown in Figure 3. Mean grain size (GS or ${\bar{D}}_{1,0}$ according with the moment-ratio notation) and Sauter’s diameter (SD or ${\bar{D}}_{3,2}$) were extrapolated from these curves. The latter parameter is widely used in several fields, in particular fluid dynamics and catalysis, where specific surface area rather than average size is the relevant parameter [51,52]. ${\bar{D}}_{3,2}$ allows to convert the multi-sized CFO grains into a monodispersed system of identical spherical CFO grains while keeping the same total area and total volume. Since in ME particulate composites the ME coupling depends on both the CFO volume fraction and CFO/PZT interphase area, ${\bar{D}}_{3,2}$ might be a helpful tool to discuss the role of the CFO microstructure.

A range of ${\bar{D}}_{3,2}$ values was obtained, from the ${\bar{D}}_{1,0}$ ≈ 70 nm/${\bar{D}}_{3,2}$ ≈ 80 nm finest narrow distribution for FM-10f, up to the ${\bar{D}}_{1,0}$ = 1.8 µm/${\bar{D}}_{3,2}$ = 3.8 µm coarsest distribution for FS-15s. Note that CFO coarsening at constant magnetic phase volume fraction increases the distance between CFO particles and therefore the PZT matrix continuity, that is, the volume of PZT free of magnetic particles (Figure 2).

### 3.2. Dielectric, Piezoelectric and Mechanical Properties

All composite materials could be poled at 4 kV mm^−1^ nominal electric field. Table 2 and Table 3 show the measured room temperature values of the piezoelectric, dielectric and elastic coefficients of the different materials with tailored microstructures. 

#### 3.2.1. Dielectric Permittivity and Loss Tangent

The relative dielectric constant of the material was determined at the frequency of 1 kHz from the measured values of the poled sample capacitance *C*_S_, also noting the loss tangent at the same frequency. 

**Figure 4:**
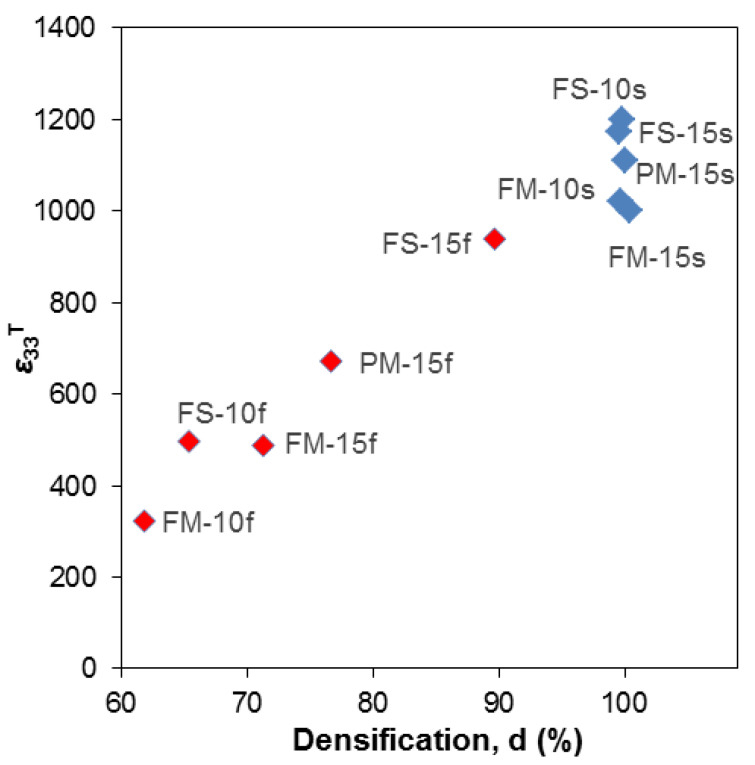
Correlation between percent density of the samples and relative dielectric constant measured at 1 kHz. Fast-sintered samples are shown as red diamonds, slow-sintered samples are shown as blue diamonds. Most low-density samples were obtained at fast sintering. Slow sintered samples mostly display the highest relative dielectric constant values.

Values were found to range between 300 and 1200, which indicates the strong sensitivity of this parameter to microstructure and the ability of obtaining a large variation of the dielectric properties of the PZT/CFO composite by changing the ceramic processing conditions. Main parameter affecting permittivity is densification, as it is shown in Figure 4 where $\varepsilon_{33}^{T}$ is displayed as a function of relative density. An obvious correlation is found, so that permittivity linearly increases with density. This is commonly observed in ceramic technologies and it is a composite effect resulting from the combination of a high dielectric material with decreasing amounts of porosity (and thus of air with a relative permittivity of 1).

**Figure 5:**
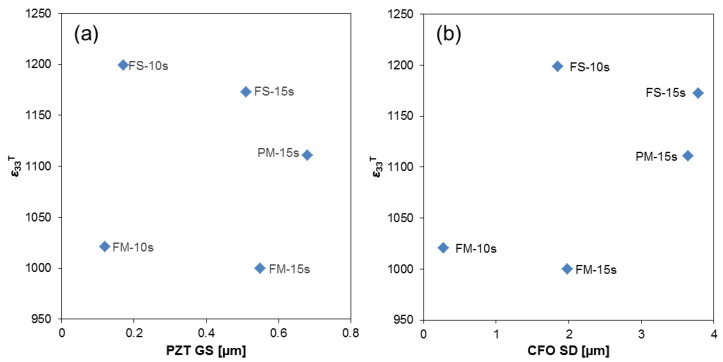
(**a**) Permittivity as a function of PZT average grain size. (**b**) Permittivity versus Sauter’s diameter of the CFO particles.

Further insight can be obtained by focusing on materials processed by slow sintering, which were all close to full densification. This allows the role of other microstructural features, such as PZT matrix grain or the CFO particle size distributions, to be addressed. Permittivity as a function of the PZT average grain size is given in Figure 5a. No trend is found and large differences between materials with analogous grain size resulted. Actually, large permittivities were consistently obtained when processing involved severe milling as compared with materials derived from powders mixed with mild milling. This suggests an effect of the CFO particle size distribution that is significantly coarsened by severe milling. Indeed, permittivity seems to roughly increase with the average diameter of the CFO particles, as shown in Figure 5b. This might be a geometrical effect but it is most probably associated with a Maxwell Wagner (M-W) type polarization because of the different conductivities of the two composite components. Mechanism is the separation of charge carriers in the CFO component as they accumulate at the CFO/PZT interfaces. It results in a step-like increase of dielectric permittivity at a given temperature (as charge carriers are thermally activated in the conductive component until exhaustion), which has associated a maximum in dielectric losses. The step position shifts towards high temperature with frequency, at the same time its height decreases. This position is determined by the charge carrier concentration and mobility of the conductive component, as well as by the length through which carriers can move before being blocked (besides by transport across the interfaces). This often results in a distinctive, yet complex dependence of permittivity on the dimension of the conductive component (the CFO particles in this case) [53]. Unlike the dielectric constant, the loss tangent at 1 kHz—whose values were found to range between 0.013 and 0.140—showed no correlation with relative density or microstructure.

#### 3.2.2. Piezoelectric Coefficients 

**Figure 6:**
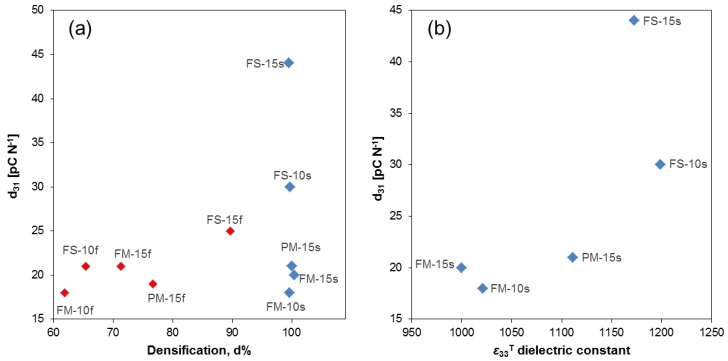
(**a**) Value of the *d*_31_ piezoelectric coefficient vs. densification (all samples). (**b**) Value of the *d*_31_ piezoelectric coefficient vs. $\varepsilon_{33}^{T}$ dielectric constant (fully densified materials only). Color coding as follows to aid the reader to evaluate correlations: red diamonds: fast sintered samples (s); blue diamonds: slow sintered samples (f).

The *d*_31_ piezoelectric coefficient is plotted vs. densification in Figure 6a below for all samples.

No trend is found and similar values were found for nanostructured materials with open porosity and fully densified ones with submicron grain size. This is a remarkable result, for nanostructured ceramics with densifications as low as 62% should be much more difficult to pole than materials with optimized PZT microstructures.

All materials present *d_31_* values between 18 and 21 pC N^−1^ but higher values were found for three ones that involved severe milling during processing. The first one is FS-15f that is the only nanostructured material with closed porosity. A value of 25 pC N^−1^ was obtained in this case, which is likely a consequence of its relatively high densification (and then permittivity). Values of 30 and 44 pC N^−1^ were attained for the FS-10s and FS-15s materials that were characterized by having large CFO particles and as shown before, resulting in high permittivity. *d_31_* as a function of permittivity is given in Figure 6b for fully densified materials. Note that behavior is not linear but exponential, which suggests large particles to highly promote poling. This is most probably a consequence of a more favorable electric field distribution within the composite and specifically across the PZT grains, as the distance between CFO grains increases.

**Figure 7:**
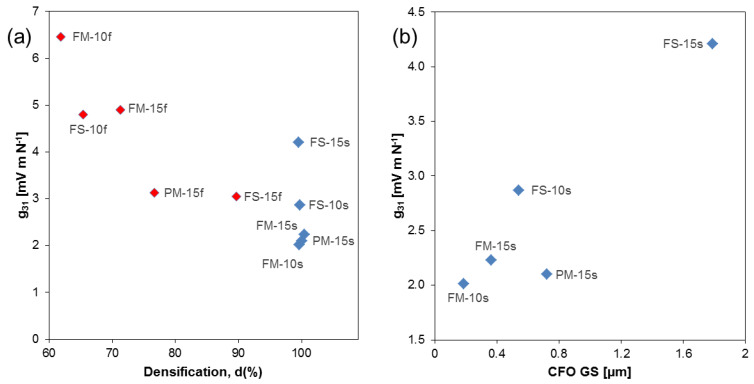
(**a**) g_31_ as a function of densification (all samples); (**b**) g_31_ vs. CFO grain size (fully densified samples only). Color coding as follows to aid the reader to evaluate correlations: red diamonds: fast sintered samples (f); blue diamonds: slow sintered samples (s).

Regarding its use in composites, the voltage piezoelectric coefficients are much more relevant than charge ones. This is so because the voltage magnetoelectric coefficients are not only proportional to the charge piezoelectric and piezomagnetic coefficients but also to the reciprocal permittivity. g*_31_* as a function of densification is given in Figure 7. Note that largest values are obtained for the nanostructured ceramics with open porosity, thanks to the ability of poling them. If one focuses only on the fully densified materials, a distinctive, roughly linear increase with the CFO GS is obtained (see Figure 7b), which proves that large particles promote poling. 

#### 3.2.3. Elastic Coefficients

Elastic coefficients were also determined as a result of the analysis of piezoresonance data. These are shown in Table 3 below.

**Figure 8:**
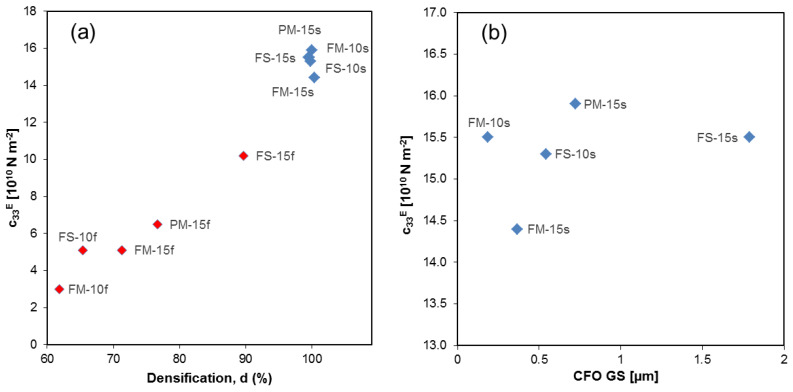
(**a**) *c*_33_^E^ vs. relative density. (**b**) *c*_33_^E^ vs. CFO grain size. Fast-sintered samples are shown as red diamonds, slow-sintered samples as blue diamonds.

Basically all mechanical parameters, namely both frequency constants *N*_p_ = *f*_R_*⋅D* and *N*_t_ = *f*_RT_⋅*t*, where *f*_R_ is the first resonance frequency in the radial mode and *f*_RT_ is the first resonance frequency in the thickness mode and *D* and *t* are the diameter and thickness of the flat disk, respectively, as well as stiffnesses *c*_33_^E^ and *c*_33_^D^, significantly correlate with percent density. This is illustrated in Figure 8 for *c*_33_^E^. Unlike permittivity and piezoelectric coefficients, when one focuses on the fully dense materials, no trend with the CFO GS is found.

### 3.3. Magnetoelectric Coefficients

**Figure 9:**
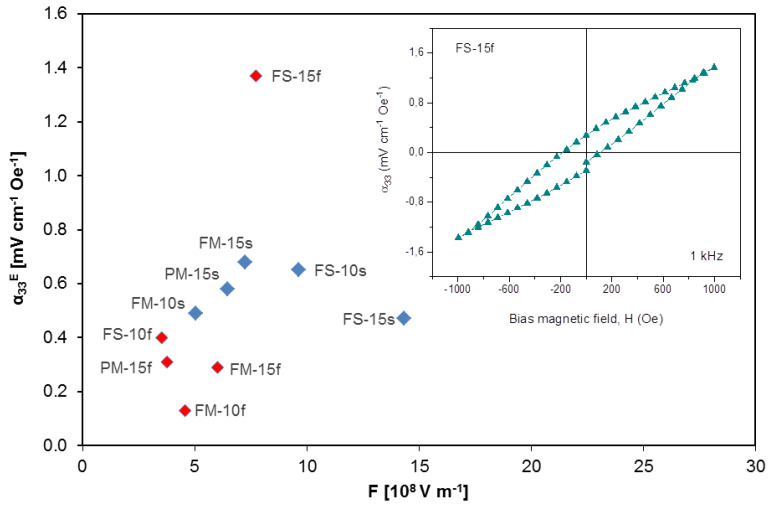
Magnetoelectric coefficient *α*_33_^E^ vs. figure of merit F (all samples). Red diamonds: fast sintered samples; blue diamonds: slow sintered samples. In the inset, an *α*_33_^E^-*H* loop for the FS-15f sample is shown between −10^3^ and +10^3^ Oe bias magnetic field.

*α_33_* magnetoelectric voltage coefficients were recorded as a function of bias magnetic field, firstly increased from 0 up to 1 kOe, then decreased from 1 down to −1 kOe and finally increased again from −1 to 0 kOe. Note that bias field for maximum response for CFO-based composites is usually higher than 1 kOe, which was the maximum field we could reach with our measuring set-up. Therefore, maximum measured values were systematically those under 1 kOe, even if they are not actual maximum ones, yet likely not far. More importantly, they allow a comparison among samples with different microstructures. A typical curve of ME coefficient as a function of H_dc_ is provided in the inset of Figure 9.

Ideally, the magnetoelectric voltage coefficient of a composite must be proportional to its voltage piezoelectric coefficient times the mechanical compliance and the piezomagnetic coefficient. One can then define a figure of merit, *F*, for the piezoelectric component as *g_33_ x c_33_^E^*. However, when one plots *α_33_* as a function of this figure of merit for all composite materials, no correlation is found (see Figure 9). This is so for fast sintered materials and for slowly heated ones. This may suggest that the magnetoeletric response is mostly controlled by the magnetostrictive response of the spinel oxide or that issue is strain transmission between components. 

Actually, when fast sintered composites are analyzed, values ranging between 0.13 and 0.4 mV/cmOe are found when open porosity exists (densification between 62 and 77%). These are the lowest magnetoelectric responses among samples and most probably reflect the poor mechanical coupling between the two phases because of large porosity. Actually, when porosity is closed as it occurs for FS-15f, an *α_33_* of 1.37 mV cm^−1^ Oe^−1^ results. 

This magnetoelectric coefficient was significantly higher than those presented by slowly heated materials with values between 0.47 and 0.65 mV cm^−1^ Oe^−1^. Nearly full densification was achieved for these composites, while densification for FS-15f was just 91%, so one would not expect mechanical coupling between components to be better in this case. Neither is it an effect of the piezoelectric matrix, because the figure of merit for FS-15f was 7.7 × 10^8^ V m^−1^, while those for the slowly heated materials spanned between 5 and 14 × 10^8^ V m^−1^. Therefore, this enhanced response is most probably associated with the magnetostrictive response of the spinel phase. This is currently not well understood and requires experimental verification but it is likely a size effect in the magnetization behavior of the CFO particles, of submicron size and actually at the threshold of the nanoscale for FS-15f but highly coarsened in the slowly heated materials [54].

## 4. Conclusions

Ten composite materials consisting of a fixed 14.4 vol% of CoFe_2_O_4_ (CFO) particles dispersed in a Nb-doped PZT matrix were produced by the solid-state method with a range of microstructures. This was accomplished by tailoring preparation through the combination of different calcination temperatures, strength of powder’s milling and sintering cycle. Fully dense composites (porosity less than 1 vol%) were obtained by conventional sintering (1100–1150 °C for 2 h), while increasing levels of porosity from closed to open configurations were introduced by fast sintering. Besides, PZT matrix nanostructuring resulted from high heating rates. Regarding the mixing/milling treatment, CFO particle coarsening was promoted by strong milling. The combination of conventional sintering at the highest temperature of 1150 °C (dense PZT matrix) and strong milling (large CFO grains in the sintered sample) led to the highest d_33_ = 96 pC/N. Actually, CFO grain growth seemed to be the most relevant microstructural feature for maximizing the piezoelectric response of fully dense magnetoelectric (ME) composites, associated with an enhanced poling of the PZT matrix. On the contrary, the highest magnetoelectric coefficient of 1.37 mV cm^−1^ Oe^−1^ was obtained when fast sintering was used instead. This material had a nanostructured PZT matrix with closed porosity and an unimodal distribution of CFO particles in the submicron range but also close to the nanoscale. Its figure of merit for ME response, *g_33_ x c_33_^E^*, was significantly lower than the previous material. This suggests a role of the magnetization behavior of the particles, so that magnetic field sensitivity decreases with coarsening.

## Figures and Tables

**Table 1:** Density (ρ), porosity (P), PbZr_x_Ti_1-x_O_3_ (PZT) lattice parameters: *a_R_*, *a_T_*, *c_T_* and *c_T_/a_T_* of rhombohedral and tetragonal perovskite (R and T, respectively), PZT and CoFe_2_O_4_ (CFO) mean grain size (GS) and CFO Sauter’s diameter (SD). Sample acronym: P = partial reaction—calcination 850 °C 4 h; F = full reaction—calcination 930 °C, 2 h; M = mild milling; S = strong milling; 10 = sintering 1100 °C; 15 = sintering 1150 °C; s = slow heating rate of 2.5 °C/min; f = fast heating rate of 44 °C/min.

Sintering	Sample			PZT Phase					CFO Phase	
	ID	ρ	P	*a_R_*	*a_T_*	*c_T_*	*c_T_/a_T_*	GS	GS	SD
		g/cm^3^	vol%	Å	Å	Å		nm	nm	nm
**Fast**	**FM** **-10f**	4.71	38	4.041	4.016	4.163	1.036	70 ± 10	73	83
	**FS** **-10f**	4.98	35	4.079	4.025	4.161	1.034	60 ± 10	79	94
	**PM** **-15f**	5.84	23	4.088	4.035	4.163	1.032	100 ± 20	110	123
	**FM** **-15f**	5.43	29	4.046	4.011	4.165	1.038	70 ± 10	114	130
	**FS** **-15f**	6.93	9	4.093	4.029	4.163	1.033	70 ± 10	75	90
**Slow**	**FM** **-10s**	7.59	0.4	4.111	4.039	4.173	1.033	120 ± 40	187	272
	**FS** **-10s**	7.6	0.2	4.107	4.031	4.17	1.034	170 ± 40	543	1848
	**PM** **-15s**	7.62	0	4.111	4.051	4.173	1.030	680 ± 150	725	3640
	**FM** **-15s**	7.65	0	4.111	4.038	4.173	1.033	550 ± 140	366	1978
	**FS** **-15s**	7.58	0.5	4.107	4.036	4.17	1.033	510 ± 80	1791	3788

**Table 2:** Electromechanical conversion factors k_p_ (planar), k_31_ and k_t_ (thickness); piezoelectric constants d_31_ and d_33_; voltage constants *g*_31_ and *g*_33_ and low-frequency dielectric constants.

Sintering	ID	k_p_	−k_31_	k_t_	−d_31_	d_33_	−g_31_	g_33_	e_33_^T^	e_33_^S^
		(−)	(−)	(−)	(pm/V)	(pm/V)	(mV m/N)	(mV m/N)	(−)	(−)
**Fast**	**FM** **-10f**	0.091	0.056	0.120	18	44	6.46	15.24	323	315
	**FS** **-10f**	0.099	0.070	0.147	21	31	4.80	6.93	497	481
	**PM** **-15f**	0.094	0.055	0.117	19	35	3.13	5.80	672	657
	**FM** **-15f**	0.107	0.064	0.121	21	51	4.90	11.81	488	475
	**FS** **-15f**	0.123	0.066	0.176	25	63	3.05	7.58	939	896
**Slow**	**FM** **-10s**	0.102	0.056	0.093	18	30	2.01	3.26	1021	1001
	**FS** **-10s**	0.161	0.086	0.144	30	67	2.87	6.31	1199	1144
	**PM** **-15s**	0.114	0.062	0.089	21	40	2.10	4.07	1111	1088
	**FM** **-15s**	0.114	0.062	0.102	20	45	2.23	5.03	1000	976
	**FS** **-15s**	0.229	0.121	0.113	44	96	4.21	9.25	1173	1097

**Table 3:** Frequency constants in the planar and thickness modes (N_p_, N_t_), mechanical stiffnesses c_33_^E^ and c_33_^D^, mechanical compliances s_11_^E^ and s_12_^E^, mechanical quality factor Q_m_, planar Poisson ratio σ^E^, acoustic wave velocity v_1_^E^ and acoustic impedance Z.

Sintering	ID	N_p_	N_t_	c_33_^E^	c_33_^D^	s_11_^E^	-s_12_^E^	Q_m_	σ^E^	v_1_^E^	Z
		(m/s)	(m/s)	(10^10^ N/m^2^)	(10^10^ N/m^2^)	(10^−12^ m^2^/N)	(10^−12^ m^2^/N)	(-)	(-)	(m/s)	(10^6^ kg/(m^2^s))
**Fast**	**FM** **-10f**	1558	1260	3.0	3.0	38.5	9.6	128	0.2498	2349	11.1
	**FS** **-10f**	1716	1516	5.1	5.2	21.0	0.3	99	0.0115	2915	16.4
	**PM** **-15f**	2060	1672	6.5	6.6	19.2	6.0	132	0.3122	2990	17.4
	**FM** **-15f**	1827	1533	5.1	5.2	25.1	6.9	112	0,2765	2711	14.7
	**FS** **-15f**	2113	1925	10.2	10.5	19.3	−9.0	104	0.4666	2735	18.9
**Slow**	**FM** **-10s**	2485	2228	15.5	15.6	11.8	4.8	145	0.4059	3377	25.0
	**FS** **-10s**	2507	2262	15.3	15.6	11.9	5.1	196	0,4309	3341	25.1
	**PM** **-15s**	2506	2287	15.9	16.0	11.4	4.7	171	0.4108	3393	25.8
	**FM** **-15s**	2490	2178	14.4	14.5	11.7	4.8	245	0.4146	3362	25.5
	**FS** **-15s**	2490	2300	15.5	15.7	12.6	5.6	1237	0.4423	3288	24.1
